# Hereditary Neuroses in Children.—IV

**Published:** 1897-06-12

**Authors:** 


### Modern Sociology.

### HEREDITARY NEUROSES IN CHILDREN.—IV.

In our last article we noticed that neurotic inheritance is in some cases associated with physical precocity. We may add that precocity of an intellectual and moral character is also met with in neurotic children, and that fond and foolish parents, who pride themselves on the premature attainments of their offspring, are apt to inflict on them much damage by a stimulating treatment, when repression would be the only wise course. As Herbert Spencer well points out,* "the abnormally rapid advance of any organ in respect of structure involves premature arrest of its growth, and this happens with the organ of the mind as certainly as with any other organ. The brain, which during early years is relatively large in mass but imperfect in structure, will, if required to perform its functions with undue activity, undergo a structural advance greater than is appropriate to its age; but the ultimate effect will be a falling short of the size and power that would else have been attained. And this is a part-cause—probably the chief cause—why precocious children, and youths who up to a certain time were carrying all before them, so often stop short, and disappoint the high hopes of their parents."

High-pressure education is, of course, specially detrimental to children of neurotic antecedents. It is this class that are likely to break down under emotional excitement and the worry of an examination in prospect. Dr. Shuttleworth has remarked that, amongst the etiological factors of mental overstrain in school life, first and foremost come a neurotic family history and a predisposition to tubercle.† It is little less than a crime to push on children with known hereditary defects to rapid brain development, for in this way (as Rachford‡ remarks) there is great risk of "developing an hereditary or acquired nervous weakness into actual disease."

In connection with the hereditary neuroses of school life, we may make special reference to chorea, which is frequently met with in over-pressed children of nervous inheritance. The Collective Investigation Committee of the British Medical Association found that more than three-fourths of the cases returned to them occurred between the ages of six and sixteen. Having certain relations with rheumatism, chorea is found frequently in phthisical families, and still more frequently in those in which there is a history of mental and nervous disorder. As regards sex, its incidence is about three times as common with girls as with boys, corresponding with the more emotional character of the former. Its onset is frequently determined by a fright or nervous shock, and the late Dr. O. Sturges was able to trace a mental cause in nearly one-half (fifty-five) of the cases he carefully investigated. The same authority found, from an analysis of the cases of twenty-five children under his care who exhibited movement disturbance due to some particular nervous overstrain, that in nine (over one-third) the exciting causes were directly connected with school, *e.g.*, overwork, punishment, preparation for examination, and so forth. Several instances were noticed by him of children with morbid infirmity of movement being punished at school for inattention or restlessness which they could not help, and he judiciously pointed out (at the Seventh International Congress of Hygiene and Demography*) the indications of incipient disorder which teachers might gather from a careful observation of the pupil's extended hand, such as might warn them against enforcing discipline likely to be productive of "school-bred chorea."

* "Education," p. 164.
† "Mental Overstrain in Education," *Lancet*, Aug. 22, 1896.
‡ "Neuroses of Childhood," p. 113.

Hereditary ataxia has been described by Friedreich as a disorder of muscular co-ordination due to arrest in the development of the spinal cord, hereditary in character, and having distinct neurotic correlations in the family history. Alcoholism has been frequently noted (by Ormerod, Everett Smith, and others) as an ancestral factor; and there is reason to believe that insane or neurotic heredity may be traced in the majority of cases. The manifestations of the disease began in some cases as early as the fourth year, a stumbling gait being one of the characteristics; and it is emphatically an affection of early life, no authentic case of onset after the twentieth year being recorded.†

Cases described by Dr. Clouston‡ and others under the designation of developmental general paralysis, but referred to by previous writers§ as cases of juvenile progressive dementia dependent upon syphilitic inheritance, call for some notice in connection with our subject, though in early childhood there may be no nervous symptoms, and the intellectual progress may be normal up to the age of the second dentition. The successive school standards are passed up to the fourth, or even higher; then it is noticed that the mental power gives way, and knowledge that has already been acquired begins to fade from the memory. The onset of the disorder is sometimes heralded by a fit or faint; and this, if attended by a fall, is often put forward by the parents as the cause of the subsequent malady. Mental impairment, muscular weakness, and deterioration of habits follow; and failure of inhibitory power is evidenced by emotional outbursts, sometimes accompanied by a torrent of terrible language, which comes as a shock from lips so juvenile. Grandiose ideas have been exceptionally noted by Clouston; but as a rule the mental state is one of inaction, with disinclination to take the trouble to recall the impressions of the past. Mental enfeeblement and muscular weakness become more intense, till at length the patient dies, a miserable example of premature senility. Clouston has traced in his cases inherited neuroses, but the more typical factor, which has been ascertained in the majority of cases recorded, is the presence of an inherited syphilitic taint.

* Transactions, vol. iv., p. 24.

† Ladame, "Brain," 1890, p. 510.

‡ "Neuroses of Development" (1888), p. 74, &c.

§ Judson Bury, "Brain," 1883; Shuttleworth, "American Journal of Insanity," January, 1888.

---

Mr. Bancroft has completed arrangements for his second three months of "Readings," on behalf of hospitals in London and throughout the country, to commence in November; and meditates a brief visit afterwards, with a like object, to the chief cities in the Dominion of Canada.

